# AAV‐Driven miR‐146a Promotes Neurite Outgrowth and Axonal Regeneration in Cortical Neurons

**DOI:** 10.1111/jnc.70372

**Published:** 2026-02-09

**Authors:** V. U. S. Matos, R. A. Almeida, C. G. Ferreira, M. T. R. Alves, M. P. Braga, M. C. Silva, T. F. da Silva, P. P. G. Guimarães, F. M. Soriani, P. Caramelli, M. R. Costa, U. Michel, V. T. Ribas

**Affiliations:** ^1^ Department of Morphology, Instituto de Ciências Biológicas Universidade Federal de Minas Gerais Belo Horizonte Brazil; ^2^ Department of Physiology and Biophysics, Instituto de Ciências Biológicas Universidade Federal de Minas Gerais Belo Horizonte Brazil; ^3^ Department of Genetics, Ecology and Evolution, Instituto de Ciências Biológicas Universidade Federal de Minas Gerais Belo Horizonte Brazil; ^4^ Behavioral and Cognitive Neurology Unit, Faculdade de Medicina Universidade Federal de Minas Gerais Belo Horizonte Brazil; ^5^ Brain Institute Universidade Federal do Rio Grande do Norte Natal Brazil; ^6^ Department of Neurology University Medical Center Göttingen Göttingen Germany

**Keywords:** AAV vector, axon regeneration, central nervous system, miR‐146a

## Abstract

Adult central nervous system (CNS) neurons exhibit limited intrinsic regenerative capacity, contributing to poor recovery after injury. MicroRNAs (miRNAs) have emerged as key regulators of many biological processes, yet their therapeutic potential in CNS repair remains incompletely understood. Here, we investigated whether adeno‐associated virus (AAV) vector‐mediated overexpression of miR‐146a enhances neurite and axon regeneration in primary cortical neurons from Wistar rats. We found that AAV.miR‐146a significantly increased neurite outgrowth, branching, and long‐distance neurite regeneration following scratch injury. Using a microfluidic platform that allows us to selectively lesion axons, we further demonstrated that AAV.miR‐146a robustly promotes axonal regrowth. Bioinformatic analyses revealed enrichment of miR‐146a target genes involved in transcriptional regulation and synaptic function, with the inflammatory adaptor TRAF6 emerging as a key predicted target. Consistent with these predictions, AAV.miR‐146a markedly reduced TRAF6 expression. Together, our results identify miR‐146a as a promising therapeutic candidate for enhancing CNS axonal repair and highlight TRAF6 signaling as a potential mechanistic link to its regenerative effects.

## Graphical abstract

In the adult central nervous system (CNS), neuronal regeneration after injury is limited, resulting in poor functional recovery. MicroRNAs (miRNAs), a class of small noncoding RNAs that control gene expression, have gained attention as potential therapeutic agents, although their contribution to CNS repair remains insufficiently understood. In this study, we demonstrate that adeno‐associated virus (AAV)‐mediated expression of miR‐146a (AAV.miR‐146a) significantly increases neurite extension and branching compared with a control AAV vector (AAV.CTRL) in uninjured rat cortical neurons. Moreover, after axonal damage, AAV.miR‐146a strongly enhances axon regeneration. Collectively, these results highlight miR‐146a as a promising molecular target to boost the regenerative capacity of CNS neurons.

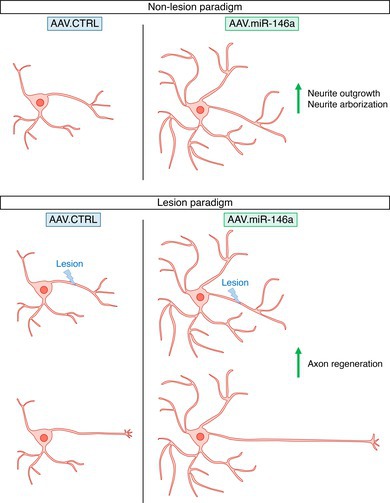

**Abbreviations**
AAV: adeno‐associated virus
ACTB: beta‐actin
ANOVA: analysis of variance
ATF3: activating transcription factor 3
cDNA: complementary DNA
CNS: central nervous system
CO₂: carbon dioxide
CREB: cAMP response element‐binding protein
CTRL: control
DAPI: 4′,6‐diamidino‐2‐phenylindole
DIV: days in vitro
DNA: deoxyribonucleic acid
E18: embryonic Day 18
EGFP: enhanced green fluorescent protein
GO: Gene Ontology
H1: RNA polymerase II/III H1 promoter
HEK‐293: human embryonic kidney 293 cells
hsa:
*Homo sapiens*

hSyn: human synapsin promoter
IL‐1R: interleukin‐1 receptor
IMG: imaging
IRAK1: interleukin‐1 receptor‐associated kinase 1
JNK: c‐Jun N‐terminal kinase
KLF4: Krüppel‐like factor 4
KLF7: Krüppel‐like factor 7
MAP 2: microtubule‐associated protein 2
MicroRNA: miRNA/miR
MTT: 3‐(4,5‐dimethylthiazol‐2‐yl)‐2,5‐diphenyltetrazolium bromide
PBS: phosphate‐buffered saline
PFA: paraformaldehyde
PNS: peripheral nervous system
PTEN: phosphatase and tensin homolog
qPCR: quantitative polymerase chain reaction
RAGs: regeneration‐associated genes
RNA: ribonucleic acid
ROCK: rho‐associated protein kinase
RRID: Research Resource Identifier (see scicrunch.org)
RT‐qPCR: real‐time quantitative polymerase chain reaction
SCI: spinal cord injury
SEM: standard error of the mean
SOCS3: suppressor of cytokine signaling 3
SOX11: SRY‐box transcription factor 11
STAT3: signal transducer and activator of transcription 3
TAK1: transforming growth factor β‐activated kinase 1
TLR: toll‐like receptor
TRAF6: tumor necrosis factor receptor‐associated factor 6
vg: viral genomes

## 1 Introduction

The adult mammalian central nervous system (CNS) exhibits a markedly limited axonal regenerative capacity, often resulting in irreversible neurological deficits after injury or in neurodegenerative diseases. The inability of adult CNS axons to regenerate after injury results from a combination of diminished intrinsic neuronal growth capacity and the presence of extrinsic inhibitory factors in the injury environment (Yiu and He 2006; Moore et al. 2009; Afshari et al. 2009). However, the limited axonal regeneration observed despite the removal of multiple extrinsic inhibitory cues underscores the critical role of intrinsic neuronal signaling in the failure of CNS regeneration (Yiu and He 2006; Sun and He 2010). Unlike the CNS, adult mammalian peripheral nervous system (PNS) exhibit a strong intrinsic ability to regenerate their axons after injury, allowing for considerable functional restoration following peripheral nerve damage (Abe and Cavalli 2008). Among the key endogenous mechanisms supporting regeneration are the activation of regeneration‐associated genes (RAGs) and the suppression of genes that inhibit axon outgrowth. Overexpression of various RAGs, including STAT3, CREB, KLF7, ATF3, SOX11, and c‐Myc, has been shown to induce axon regeneration in the CNS (Ribas and Costa 2017). Conversely, downregulation of inhibitory genes such as PTEN, SOCS3, KLF4, ROCK, and RhoA can also promote axonal regrowth (Ribas and Costa 2017). Because many of these genes encode proteins that control transcription, strategies aimed at modulating the expression of molecules that control gene expression hold significant potential for promoting axonal regeneration.

Recently, the importance of microRNAs (miRNAs) in axon regeneration has been highlighted in different studies (Wang et al. 2020; Li et al. 2021; Hintermayer et al. 2024; Chen et al. 2024; Shoukeer et al. 2024; Shen et al. 2024). MiRNAs are small, non‐coding single‐stranded RNAs that act as key regulators of gene expression at the post‐transcriptional level including within the CNS (Bushati and Cohen 2007). In the nervous system they have been implicated in the control of different biological processes during development including neural patterning, cell specification, axonal pathfinding and apoptosis, and in mature neurons such as synaptic plasticity (Kosik 2006). Recent studies have also reported dysregulation of miRNA expression in the pathogenesis of neurodegenerative diseases, highlighting their potential as both biomarkers and therapeutic targets (Azam et al. 2024; Ueno et al. 2024; Ramirez‐Gomez et al. 2025; Duarte et al. 2025).

Among the miRNAs enriched in the CNS, miR‐146a ranks among the most abundant and shows clear dysregulation across multiple neurological disorders (Fan et al. 2020). As a potential therapeutic target, miR‐146a functions to suppress signaling cascades associated with inflammation (Aslani et al. 2021). MiR‐146a is also essential for CNS development, as its deficiency disrupts radial glial cell differentiation, impairs neurogenesis, and restricts neurite outgrowth (Fregeac et al. 2020). Evidence also shows that miR‐146a is enriched in the axons of dorsal root ganglion neurons (Jia et al. 2016). Furthermore, gain‐ and loss‐of‐function experiments in these neurons in vitro demonstrate that miR‐146a, by regulating its target genes IRAK1 and TRAF6, modulates axonal growth (Jia et al. 2016). Collectively, these findings suggest that miR‐146a represents a promising target for promoting CNS repair. However, its precise role in CNS axonal regeneration remains to be elucidated. Building on the evidence of miR‐146a's involvement in axon growth in the PNS and in CNS development, the present study aims to investigate whether overexpression of miR‐146a via recombinant adeno‐associated viral (AAV) vectors can enhance axon and neurite regeneration in CNS neurons.

## 2 Materials and Methods

### 2.1 Cloning and Production of AAV Vectors

The AAV.miR‐146a vector expresses the miR‐146 precursor (pre‐miR‐146) under the control of the RNA polymerases II/III H1 promoter (Gao et al. 2018) and the green fluorescent protein (EGFP) under the control of the human synapsin (hSyn) promoter as a reporter gene (AAV.miR‐146a). As a control we used an AAV vector that expresses only EGFP under the control of the hSyn promoter (AAV.CTRL).

For all experiments, we used the AAV pseudotype 1/2, which consists of an AAV2‐derived genome packed into hybrid capsids of AAV1 and a mutated form of the AAV2 capsid (Balke et al. 2020). AAV were produced by transfecting HEK‐293 cells (RRID: CVCL_0045) with pAAV.miR‐146a or pAAV.CTRL plasmids plus the helper plasmids. AAV purification was done by iodixanol gradient ultracentrifugation and fast protein liquid chromatography. Purified AAV vectors were tested on primary cortical neurons to evaluate transduction efficacy and toxicity. Viral titers were quantified by qPCR and expressed as viral genome (vg) per microliter (μL) (AAV.miR‐146a: 1.7 × 10e8 vg/μL; AAV.CTR: 1.6 × 10e8 vg/μL).

### 2.2 Primary Culture of Cortical Neurons

Primary cortical neurons were isolated from Wistar rat embryos at embryonic Day 18 (E18) ( *Rattus norvegicus* , Wistar strain; RRID: RGD_13508588) provided by Central Animal Facility of UFMG (https://www.ufmg.br/prpq/bioterio/), following previously described protocols (Ribas et al. 2021; Almeida et al. 2024) with the approval of the Animal Use Ethics Committee of the Federal University of Minas Gerais (number 237/2018). In brief, pregnant Wistar rats (total number: 18) were euthanized by carbon dioxide asphyxiation followed by cervical dislocation to ensure death, after which the embryos were collected and euthanized by decapitation. Embryonic cortices were dissected, pooled, enzymatically dissociated using trypsin (Sigma‐Aldrich; cat. no. T4549), and mechanically triturated with a custom Pasteur pipette. Following centrifugation, neurons were resuspended in cortex medium consisting of serum‐free neurobasal medium (Thermo Fisher Scientific; cat. no. 21103049) supplemented with B‐27 (Thermo Fisher Scientific; cat. no. 17504044), GlutaMAX (Thermo Fisher Scientific; cat. no. 35050061), penicillin/streptomycin/neomycin (Thermo Fisher Scientific; cat. no. 15640055), and transferrin (Sigma‐Aldrich; cat. no. T8158). Neurons were plated onto 24‐well plates pre‐coated with poly‐L‐ornithine (Sigma‐Aldrich; cat. no. P3655) and laminin (Sigma‐Aldrich; cat. no. L2020) or in appropriate culture dishes, and maintained at 37°C under 5% CO_2_. Culture medium was refreshed every other day.

### 2.3 Transduction of Primary Cortical Neurons With AAV Vectors and Cell Viability Assay

AAV transduction was performed 4 h after plating using AAV.CTRL or AAV.miR‐146a vectors at the titer of 1 × 10^7^ vg/well, which produced minimal toxicity and comparable transduction efficiencies of 80%–90% at day in vitro (DIV) 8, as determined by EGFP fluorescence using an Axio Vert.A1 KMAT microscope (Zeiss; RRID: SCR_022030) with ZEN software (RRID: SCR_013672). Cell viability was also assessed on DIV8 post‐transduction using alamar Blue assay (Thermo Fisher Scientific; cat. no. DAL1025) and measured with a Cytation 5 plate reader (Agilent Biotek; RRID: SCR_019732), following manufacturer's instructions.

### 2.4 Neurite Outgrowth Assay and Quantification

To assess neurite outgrowth in vitro, cortical neurons were plated on glass coverslips in 24‐well plates at a density of 80 000 cells per well and transduced with either AAV.CTRL or AAV.miR‐146a vectors. Two coverslips per vector were used for each culture. On DIV8, neurons were fixed with 4% paraformaldehyde (PFA; Sigma‐Aldrich; cat. no. P6148) in phosphate‐buffered saline (PBS; Thermo Fisher Scientific; cat. no. 10010023) and immunostained with an antibody against the cytoskeletal protein β‐III‐tubulin (Tuj‐1; BioLegend; cat. no. 801202). For each coverslip, 10 random fields were captured at 20× magnification using a Zeiss Axio Imager Apotome.2 microscope (RRID: SCR_018876) with ZEN software. Neurite outgrowth was quantified in a blinded manner using ImageJ software (RRID: SCR_003070) by measuring the area stained for Tuj1 and normalizing it to the total number of cells per image as previously reported (Almeida et al. 2024).

### 2.5 Neurite Arborization Assessment

The complexity of neurite branching was evaluated using Sholl analysis on individual neurons. Neurons were plated at a low density (20 000 cells/well) on glass coverslips in 24‐well plates, transduced with AAV.CTRL or AAV.miR‐146a, fixed on DIV8, and immunostained with Tuj‐1 as described above. Two coverslips per vector were analyzed per culture. At least 10 isolated neurons per coverslip were imaged at 40× magnification using the Zeiss Axio Imager Apotome.2. Sholl analysis was performed using ImageJ with the Neuroanatomy plugin. Concentric circles were drawn around the cell soma, starting 5 μm from the center and extending to 50 μm, with 5 μm intervals. The number of neurite intersections per circle and the total intersections were quantified in a blinded manner to evaluate neurite arborization complexity. In addition to Sholl analysis, total neurite length per neuron was also quantified using ImageJ with the NeuronJ plugin.

### 2.6 Mechanical Scratch Assay and Quantification of Neurite Regeneration

Neurite regeneration was assessed using a mechanical scratch model. Neurons were seeded at 100 000 cells per well on glass coverslips in 24‐well plates and transduced with AAV.CTRL or AAV.miR‐146a. On DIV7, a linear scratch was made across the center of each coverslip using a pipette tip. Twenty‐four hours post‐lesion, neurons were fixed and immunostained with Tuj‐1 antibody. Two coverslips per vector were analyzed per culture. At least five images per coverslip were acquired at 20× magnification in the center of the scratch. Regenerating neurites were traced using ImageJ with the NeuronJ plugin. Quantification included the total neurite length within 100–200 μm from the scratch edge and the number of neurites crossing a line positioned 200 μm from the scratch. All measurements were normalized to the number of cells within 100 μm of the lesion border.

### 2.7 Culture of Cortical Neurons in Microfluidic Chambers

Microfluidic chambers (Park et al. 2006) were prepared as described previously (Ribas et al. 2021; Almeida et al. 2024). Each chamber consists of two main channels (“somatic” and “axonal”) connected by microgrooves, allowing physical separation of axons from cell bodies and dendrites. Chambers were mounted on 35 mm glass coverslips (Fisher‐Scientific; cat. no. NC1454455) coated with 0.1 mg/mL poly‐L‐lysine (Sigma‐Aldrich; cat. no. P4707). Primary cortical neurons were seeded into the somatic compartment at 4 × 10^5^ cells per chamber and transduced with AAV.CTRL or AAV.miR‐146a 4 h later. Medium in both compartments was partially replaced every other day, with a larger volume in the somatic compartment to direct axonal growth through the microgrooves.

### 2.8 Axotomy and Quantification of Axonal Regeneration

After DIV7–9, once axons reached the axonal compartment, axotomy was performed by vacuum aspiration of the culture medium from this compartment, followed by replacement with fresh cortex medium. Axotomy was confirmed using transmitted light microscopy with an Axio Vert.A1 KMAT microscope. Forty‐eight hours post‐lesion, neurons were fixed and immunostained with Tuj‐1. Regenerating axons were imaged using a 10× objective. The Tile function in ZEN software was used to capture the entire axonal compartment along 25 central microgrooves. Regenerating axons were quantified at distances of 500 and 1000 μm from the lesion in a blinded manner.

### 2.9 Immunofluorescence

Neurons were fixed in 4% PFA for 10 min, washed with PBS, and permeabilized with 0.5% Triton X‐100 (Sigma‐Aldrich; cat. no. 93443) in PBS for 20 min. Cells were blocked in PBS containing 5% normal goat serum (Sigma‐Aldrich; cat. no. G9023) and 1% bovine serum albumin (Sigma‐Aldrich; cat. no. A7030) for 1 h at room temperature, then incubated overnight at 4°C with Tuj1 (β‐III‐tubulin) primary antibody (1:10 000) in blocking solution. β‐III‐tubulin detection was performed with Alexa Fluor 546‐conjugated secondary antibody (1:1000; Thermo‐Scientific; cat. no. A11030) for 1 h at room temperature. Nuclei were counterstained with DAPI (Thermo Fisher Scientific; cat. no. D1306), and slides were mounted with Fluoromount‐G Mounting Medium (Thermo‐Scientific; cat. no. 00‐4958‐02) and stored at 4°C until imaging.

### 2.10 Prediction of miRNA Targets

To explore the potential mechanisms through which miR‐146a may influence axonal regeneration, computational predictions of miR‐146a target mRNAs were performed. Target prediction for the mature form of human miR‐146a (hsa‐miR‐146a‐5p) was carried out using miRWalk version 3.0 (RRID: SCR_016509) (Sticht et al. 2018). Experimentally validated miRNA–mRNA interactions were retrieved from the integrated miRTarBase database (RRID: SCR_017355) (Huang et al. 2022), which compiles verified miRNA targets from the literature using natural language processing. Only interactions with a probability score ≥ 0.8 were retained to ensure a high confidence level in the predicted targets.

### 2.11 Gene Ontology (GO) Enrichment Analysis

GO analysis for the 112 predicted miR‐146a targets (Table S1) was performed using the EnrichR package (RRID: SCR_001575) (Kuleshov et al. 2016), available at https://cran.r‐project.org/web/packages/enrichR/vignettes/enrichR.html. GO enrichment was calculated for the databases “GO_Molecular_Function_2025,” “GO_Cellular_Component_2025,” “GO_Biological_Process_2025,” “TISSUES_Experimental_2025,” and “SynGO_2024.”

### 2.12 Real Time Quantitative PCR (RT‐qPCR)

The expression of mRNAs was evaluated by qPCR. Neurons were plated at 500 000 cells/well in 24‐well plates, transduced with AAV.CTRL or AAV.miR‐146a and on DIV8 the RNA was extracted. Total RNA was obtained from primary neuron culture using GeneJET RNA Purification Kit (Thermo Fisher Scientific; cat. no. K0731) according to the manufacturer's instructions. One microgram of RNA was used to synthesize first strand cDNA, prepared with High‐capacity cDNA reverse transcription kit (Applied Biosystems; cat. no. 4368814) following the manufacturer's protocol. Quantitative PCR was performed using PowerTrack SYBR Green Master Mix (Applied Biosystems; cat. no. A46109) on a QuantStudio 3 Real‐Time PCR System (Applied Biosystems; RRID: SCR_020238). The primer sequences used were: TRAF6 (Forward: GTAGCCTCCACTTACCTGTG—Reverse: TGTAATGTGACCTCGCTGATG), and ACTB (Forward: AGGGAAATCGTGCGTGACAT—Reverse: AATGCCTGGGTACATGGTGG). The reaction was performed in biological triplicates and technical replicates; the relative expression was normalized by the constitutive gene ACTB using the $2^{-∆∆C_{t}}$ method.

### 2.13 Statistical Analysis

Statistical analyses were conducted using GraphPad Prism software (version 9.5.1). Data normality was assessed using the Shapiro–Wilk test prior to the application of parametric analyses. Sample sizes were not established through a priori statistical calculations. Instead, the number of samples used in each experiment was guided by previously published studies employing similar experimental paradigms, in which the selected sample sizes provided adequate statistical power to identify biologically meaningful effects (Ribas et al. 2021; Almeida et al. 2024). Outliers were assessed using the ROUT (Robust regression and Outlier removal) method from GraphPad Prism software, and no outliers were identified in the dataset. Data were analyzed using *t*‐test or two‐way ANOVA, followed by Sidak multiple comparison test. Data were presented as mean ± standard error of the mean (SEM). The level of significance was set at *p* ≤ 0.05.

## 3 Results

### 3.1 AAV.miR‐146a Efficiently Transduce Primary Cortical Neurons

**FIGURE 1:**
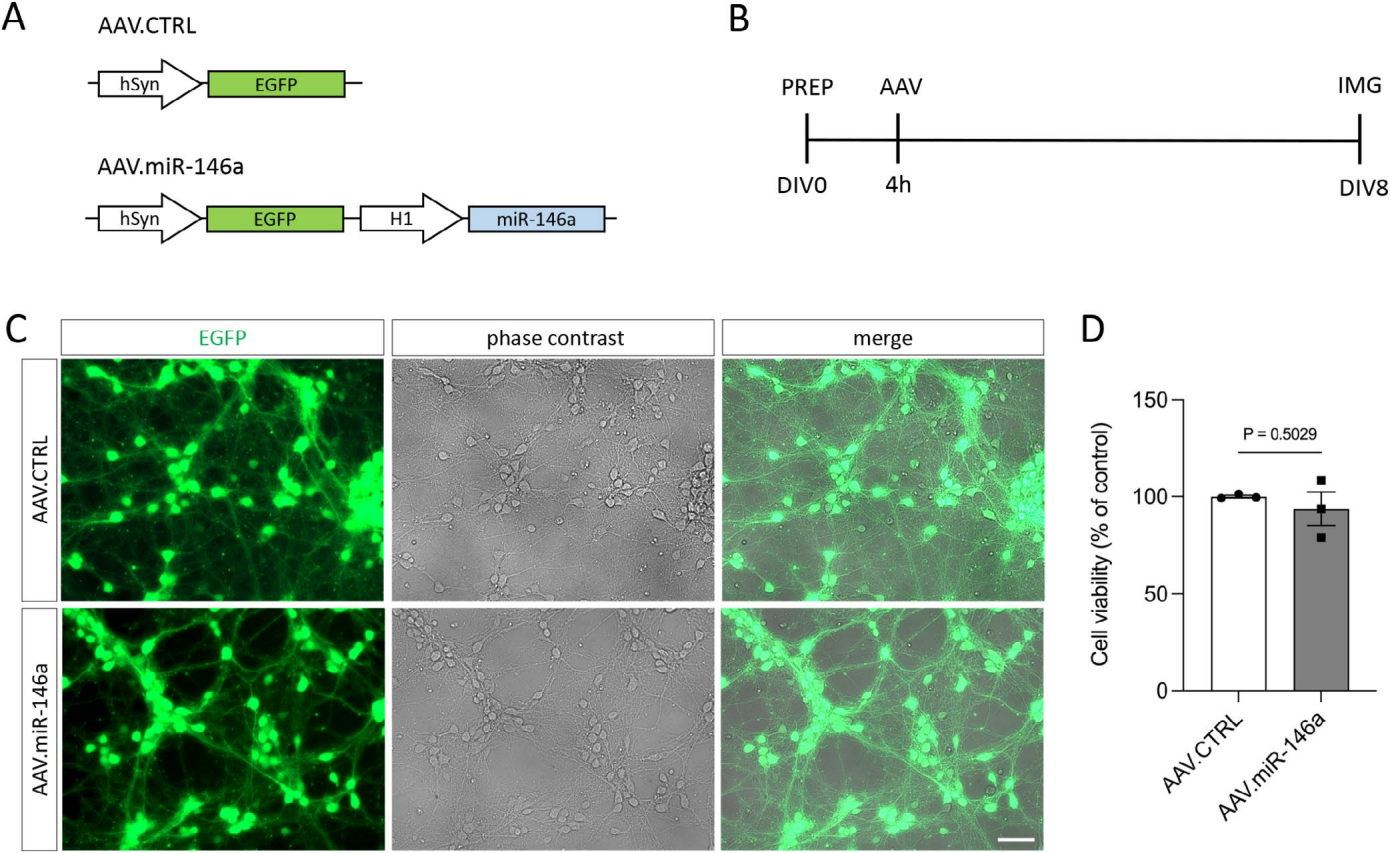
Transduction of primary cortical neurons with AAV.miR‐146a or AAV.CTRL. (A) Schematic representation of the AAV constructs used for neuronal transduction. The control vector (AAV.CTRL) and the miR‐146a‐expressing vector (AAV.miR‐146a) both encode the fluorescent reporter EGFP driven by the human synapsin (hSyn) promoter, whereas miR‐146a expression is regulated by the H1 promoter. (B) Experimental timeline for assessing AAV transduction efficiency. PREP, preparation of E18 cortical neurons and plating (150 000 cells/well); AAV, viral transduction; DIV, days in vitro; IMG, imaging time point. (C) Representative fluorescence and phase‐contrast images of cortical neurons at DIV8 following transduction with either AAV.CTRL or AAV.miR‐146a, showing robust EGFP expression (green). Scale bar: 50 μm. (D) Assessment of neuronal viability at DIV8 (*n* = 3 independent cultures). Data are shown as single data points with mean ± SEM. No significant difference was detected between groups. Two‐tailed unpaired *t*‐test, *t* = 0.7354, df = 4, *p* = 0.5029.

We aimed to test whether miR‐146a expression modulates neurite/axon outgrowth and regeneration of primary cortical neurons in vitro. For that, miR‐146a overexpression was induced by AAV vector expressing the miR‐146a under the control of the H1 promoter together with the EGFP under the control of the hSyn (AAV.miR‐146a). The control vector only expresses the EGFP under the control of the hSyn promoter (AAV.CTRL) (Figure 1A). First, we evaluated if the AAV.miR‐146a and AAV.CTRL were able to transduce primary cortical neurons. Neurons were plated, transduced with the AAV vector and on DIV8 imaged using a fluorescence microscope. We found that AAV.miR‐146a and AAV.CTRL were both able to transduce efficiently primary cortical neurons with around 90% of the cells expressing EGFP (Figure 1B,C). We also tested for cytotoxicity and did not find differential cytotoxicity between the AAV.miR‐146a and AAV.CTRL vectors (Figure 1D). Thus, we found that both AAV vectors efficiently transduce rat primary cortical neurons with no difference in toxicity.

### 3.2 AAV.miR‐146a Promotes Neurite Outgrowth and Branching

**FIGURE 2:**
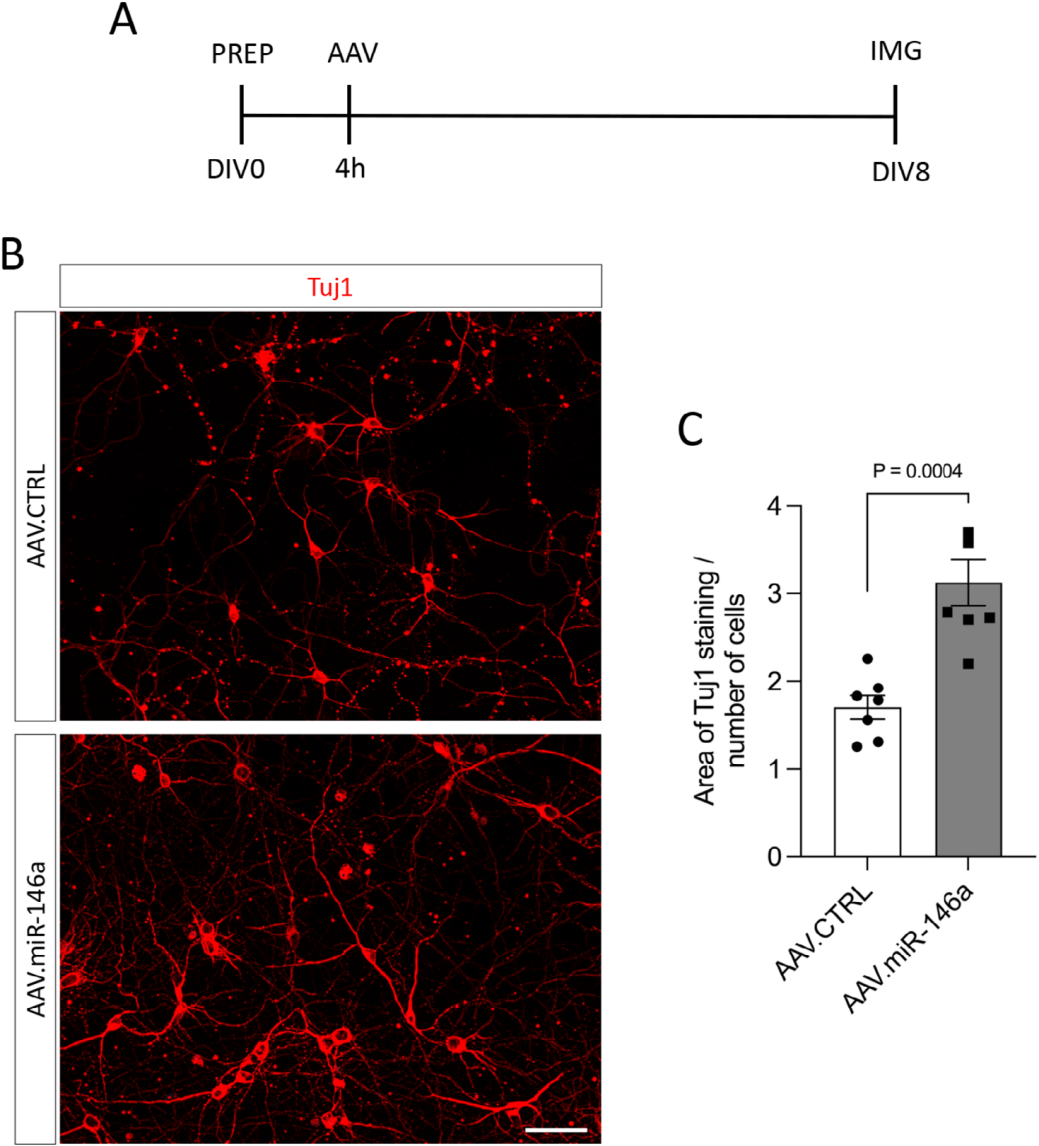
AAV.miR‐146a promotes neurite outgrowth. (A) Experimental timeline for the neurite outgrowth assay. PREP, preparation of E18 cortical neurons and plating (80 000 cells/well); AAV, viral transduction; DIV, days in vitro; IMG, imaging time point. (B) Representative fluorescence images of DIV8 cortical neurons transduced with either AAV.miR‐146a or AAV.CTRL and immunostained for β‐III‐tubulin (Tuj1, red). Scale bar: 50 μm. (C) Quantification of the Tuj1‐positive area normalized to the number of neuronal cell bodies (*n* = 7 independent cultures). Data are shown as single data points with mean ± SEM. Two‐tailed unpaired *t*‐test, *t* = 4.811, *p* = 0.0004.

To investigate whether AAV.miR‐146a affects neurite extension, primary cortical neurons were transduced with either AAV.miR‐146a or control (AAV.CTRL) vectors. After eight days in culture, neurons were fixed and immunolabeled for the cytoskeletal marker β‐III‐tubulin (Tuj1) to visualize the neurons (Figure 2A,B). The Tuj1‐positive area was quantified by fluorescence microscopy and normalized by the number of neurons per field. We found that neurons transduced with AAV.miR‐146 showed increased Tuj1‐stained area in comparison to neurons transduced with AAV.CTRL (AAV.miR‐146: 3.1 ± 0.26; AAV.CTRL: 1.7 ± 0.13) (Figure 2C), suggesting that AAV.miR‐146a can promote neurite outgrowth.

**FIGURE 3:**
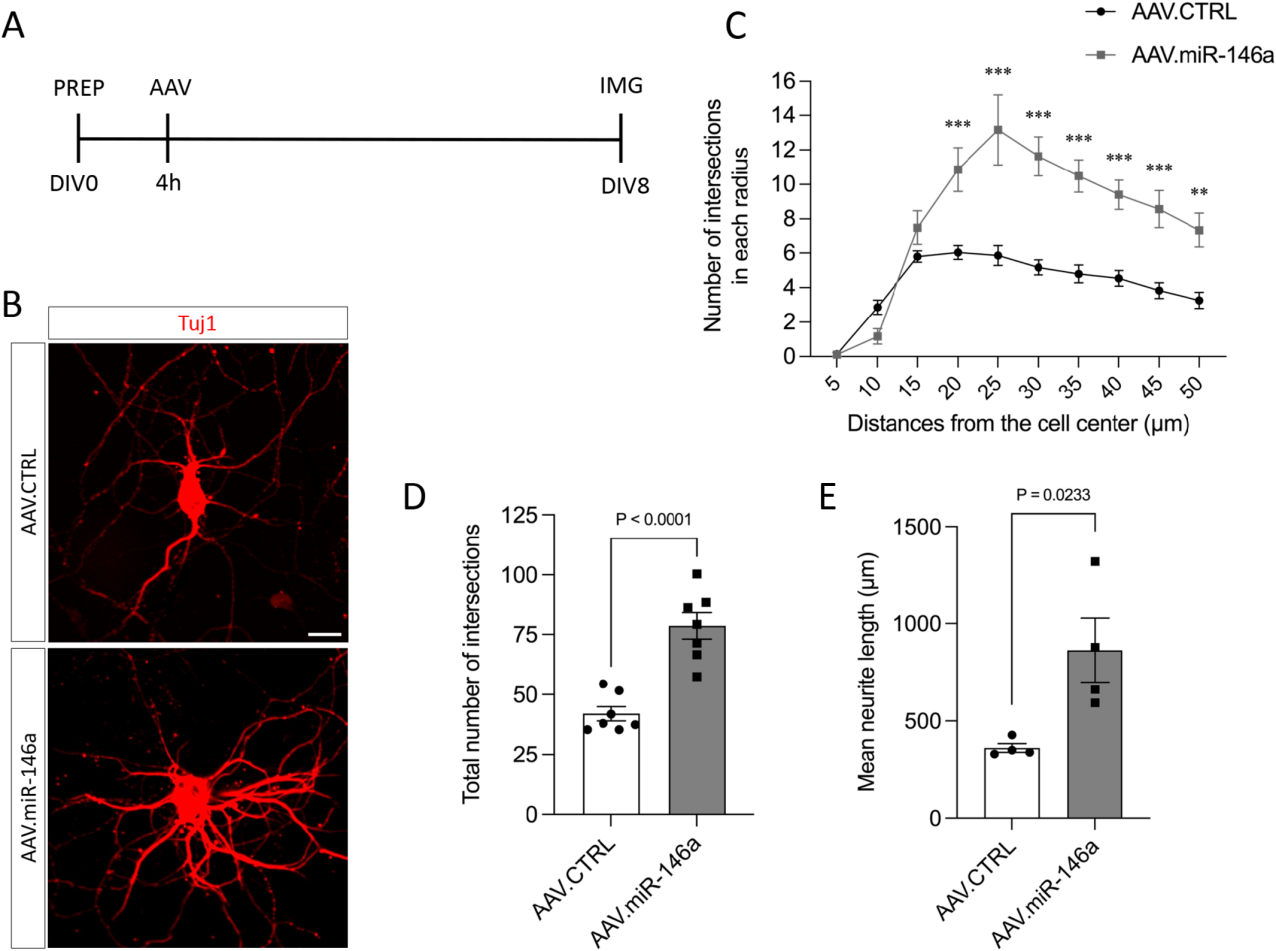
The complexity of the neurite arborization is increased by AAV.miR‐146a. (A) Experimental design for the assessment of neurite branching complexity. PREP, preparation of E18 cortical neurons and plating (20 000 cells/well); AAV, viral transduction; DIV, days in vitro; IMG, imaging time point. (B) Representative fluorescence images of isolated DIV8 cortical neurons transduced with either AAV.miR‐146a or AAV.CTRL and immunostained for β‐III‐tubulin (Tuj1, red) Scale bar: 20 μm. (C) Quantification of neurite intersections with concentric circles at increasing radial distances from the soma (*n* = 7 independent cultures). Data are expressed as mean ± SEM. Two‐way ANOVA (Radii: *F* = 25.42, *p* < 0.0001; AAV vector: *F* = 104.4, *p* < 0.0001; interaction: *F* = 6068, *p* < 0.0001), followed by Sidak multiple comparison test **p < 0.01, ***p < 0.001. (D) Total number of intersections across all circles (*n* = 7 independent cultures). (E) Quantification of mean neurite length per neuron (*n* = 4 independent cultures). (D, E) Data are presented as single data points and mean ± SEM. Two‐tailed unpaired *t*‐test (D) *t* = 5.831, *p* < 0.0001 and (E) *t* = 3.023, *p* = 0.0233.

The potential impact of AAV.miR‐146a on neurite branching was also examined. For this purpose, neurons were plated at low density to allow single‐cell evaluation, transduced and immunostained as above, and subjected to Sholl analysis to assess arborization patterns (Figure 3A). The number of neurite intersections was significantly higher at the distances from 20 to 50 μm in the AAV.miR‐146a transduced neurons compared with the control (Figure 3B,C). Likewise, the cumulative number of intersections across all radii was significantly higher in neurons transduced with AAV.miR‐146a (70 ± 6.5) compared with AAV.CTRL (42 ± 3.0) (Figure 3D). Additionally, we evaluated total neurite length per neuron and found that transduction with AAV.miR‐146a (863 μm ± 164) significantly enhances neurite outgrowth compared with AAV.CTRL (362 μm ± 22). Collectively, these findings indicate that forced expression of miR‐146a promotes neurite outgrowth and enhances the complexity of neurite arborization in cultured cortical neurons.

### 3.3 AAV.miR‐146a Promotes Neurite Regeneration After Scratch Lesion

**FIGURE 4:**
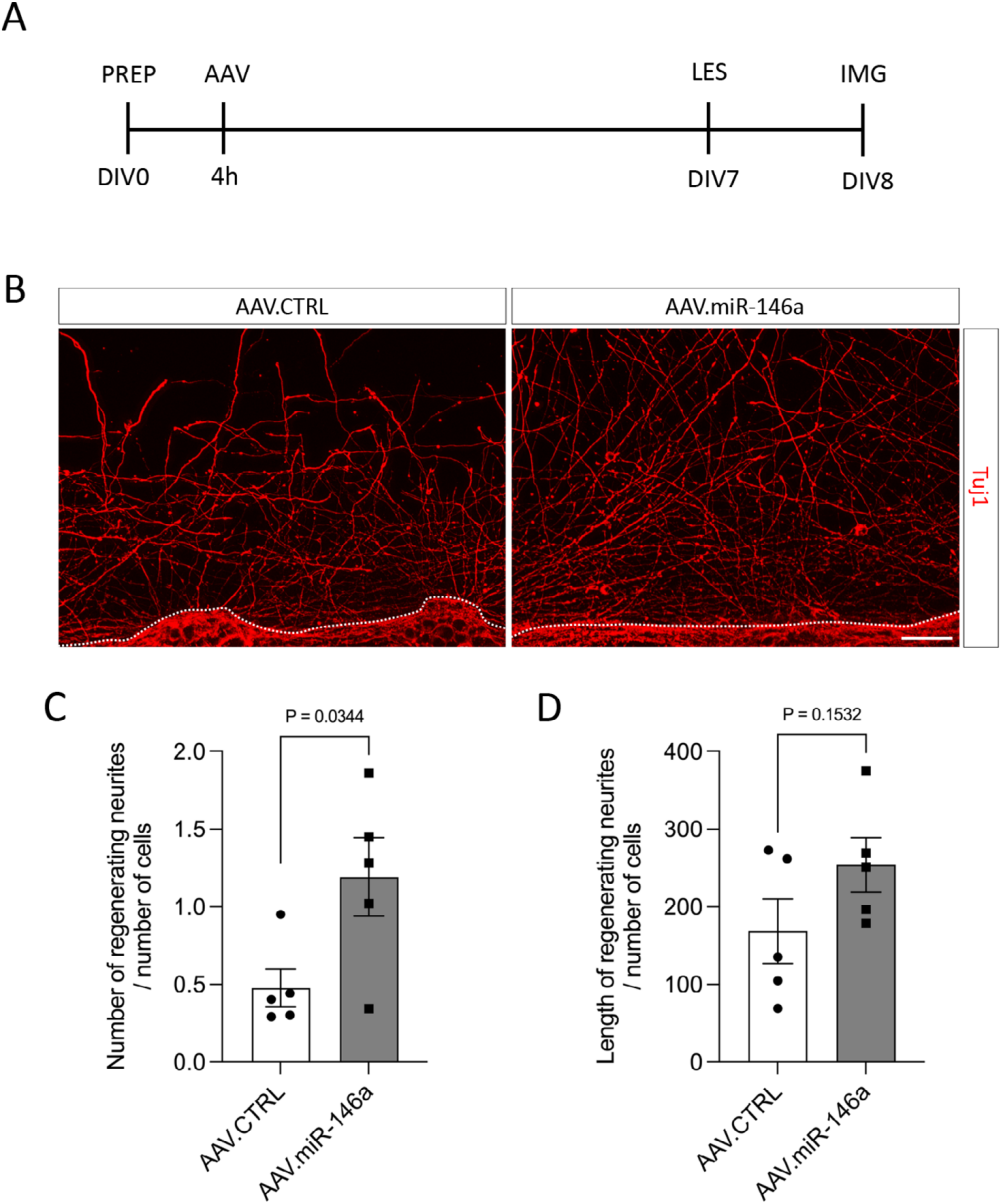
AAV.miR‐146a promotes neurite regeneration following scratch injury. (A) Experimental workflow for the neurite regeneration assay. PREP, preparation of E18 cortical neurons and plating (100 000 cells/well); AAV, viral transduction; DIV, days in vitro; IMG, imaging; LES, induction of the scratch lesion. (B) Representative fluorescence images of cortical neurons transduced with either AAV.CTRL or AAV.miR‐146a and fixed 24 h after the scratch lesion, showing regenerating neurites labeled with β‐III‐tubulin (Tuj1, red). The dashed line marks the lesion border. Scale bar: 50 μm. (C) Quantification of total neurite length within the lesion zone at a distance of 100–200 μm from the scratch border. (D) Quantification of the number of neurites extending to 200 μm beyond the lesion border. Data represented as single data points and mean ± SEM (*n* = 5 independent cultures). Two‐tailed unpaired *t*‐test, (C) *t* = 2.546, *p* = 0.0344 and (D) *t* = 1.578, *p* = 0.1532.

After demonstrating that AAV.miR‐146a enhances neurite outgrowth and branching, we next investigated whether it could also promote neurite regrowth following CNS injury. To test this, we employed an in vitro scratch‐lesion model. Primary cortical neurons were transduced with either AAV.miR‐146a or AAV.CTRL, and a mechanical scratch was applied 7 days later. Twenty‐four hours after the lesion, neurons were fixed, immunostained with Tuj1 antibody, and neurite regeneration within the lesion area was quantified by measuring neurite length and number (Figure 4A). Within the area between 100 and 200 μm, no significant difference in total neurite length was detected between AAV.CTRL and AAV.miR‐146a groups (169 ± 42 vs. 254 ± 35) (Figure 4B,C). In contrast, for neurites extending ≥ 200 μm beyond the lesion border, AAV.miR‐146a significantly increased the number of regenerating neurites normalized by the number of cells compared with control (1.19 ± 0.25 vs. 0.48 ± 0.12) (Figure 4B,D). These findings indicate that miR‐146a overexpression enhances long‐distance neurite regeneration in vitro following a CNS‐like injury.

### 3.4 AAV.miR‐146a Enhances Axon Regeneration Following Axotomy

**FIGURE 5:**
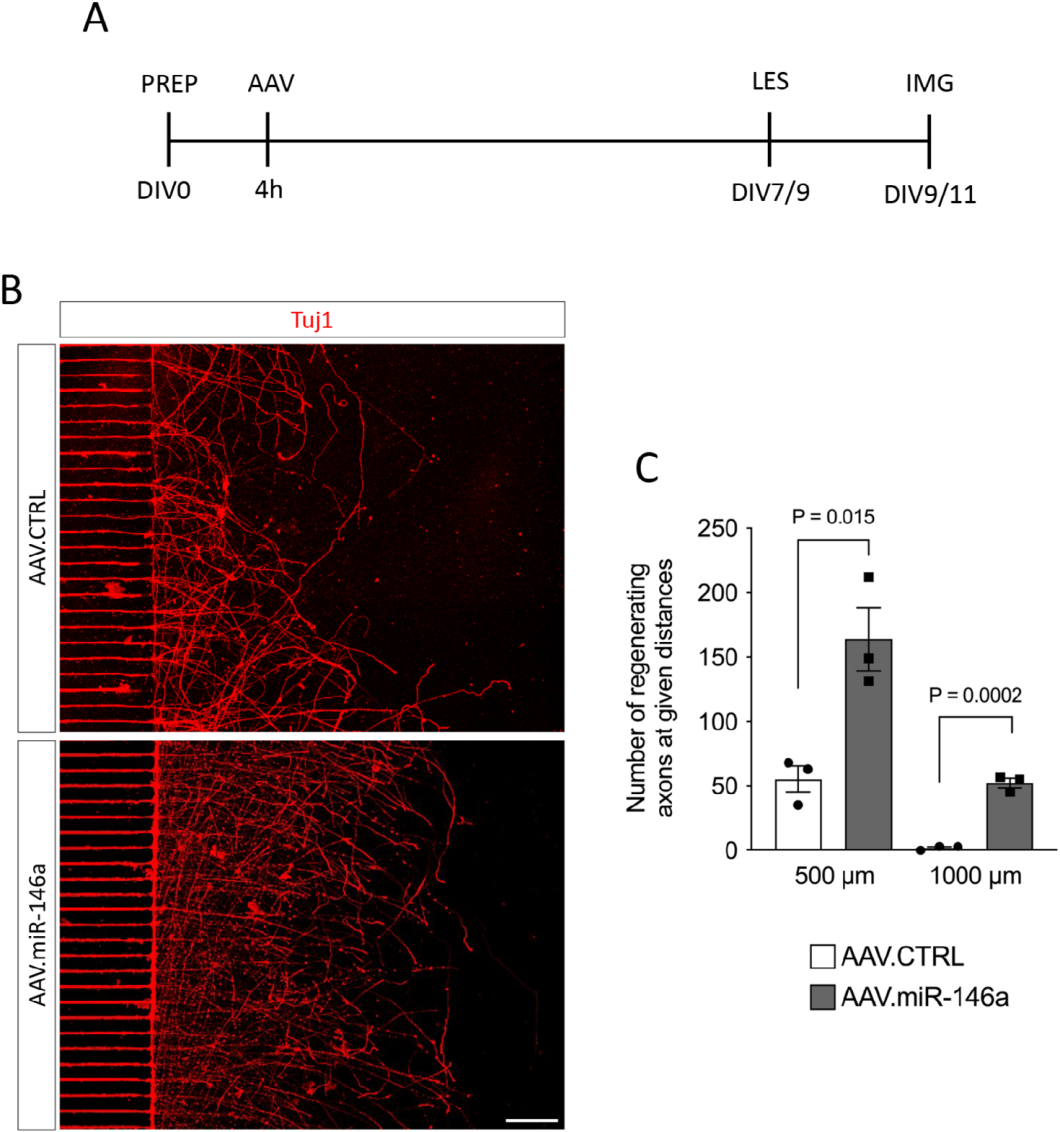
Axonal regrowth after axotomy is enhanced by AAV.miR‐146a. (A) Experimental design for the assessment of axonal regeneration. PREP, preparation of E18 cortical neurons and plating in microfluidic devices (400 000 cells/device); AAV, viral transduction; DIV, days in vitro; IMG, imaging; LES, induction of axotomy. (B) Representative fluorescence images of the microgrooves (left) and axonal compartment (right) showing regenerating axons labeled with β‐III‐tubulin (Tuj1, red) of cortical neurons transduced with either AAV.CTRL or AAV.miR‐46a, fixed 48 h after axotomy. Scale bar: 200 μm. (C) Quantification of number of axons at the given distances from microgroove exit. Data represented as single data points and mean ± SEM (*n* = 3 independent cultures). Two‐tailed unpaired *t*‐test for each distance, 500 μm: *T* = 4.083, *p* = 0.015, 1000 μm: *T* = 13.09, *p* = 0.0002.

Because the scratch‐lesion assay does not allow clear distinction between axons and dendrites, we employed a previously established model of selective axonal injury using microfluidic devices (Ribas et al. 2021; Almeida et al. 2024). This system physically separates dendrites from axons, enabling targeted axotomy and precise assessment of axonal regeneration without dendritic interference. Primary cortical neurons were seeded into the microfluidic chambers and transduced with either AAV.miR‐146a or AAV.CTRL. After 7–9 days in vitro, when axons had extended through the microgrooves to reach the axonal compartment, a selective axotomy was performed. Forty‐eight hours later, cultures were fixed and immunostained with β‐III‐tubulin (Tuj1) to evaluate axonal regeneration (Figure 5A). Neurons transduced with AAV.miR‐146a exhibited a marked increase in the number of regenerating axons compared with controls, with significant differences observed at both 500 (164 ± 25 vs. 55 ± 10) and 1000 μm (52 ± 4 vs. 2 ± 1) from the lesion site (Figure 5B,C). These findings indicate that miR‐146a specifically enhances axonal regeneration following axotomy. Collectively, these results demonstrate that, beyond promoting neurite outgrowth and regeneration, AAV.miR‐146a also drives robust axonal regrowth in vitro.

### 3.5 AAV.miR‐146a Downregulates the Expression of TRAF6 in Cortical Neurons

**FIGURE 6:**
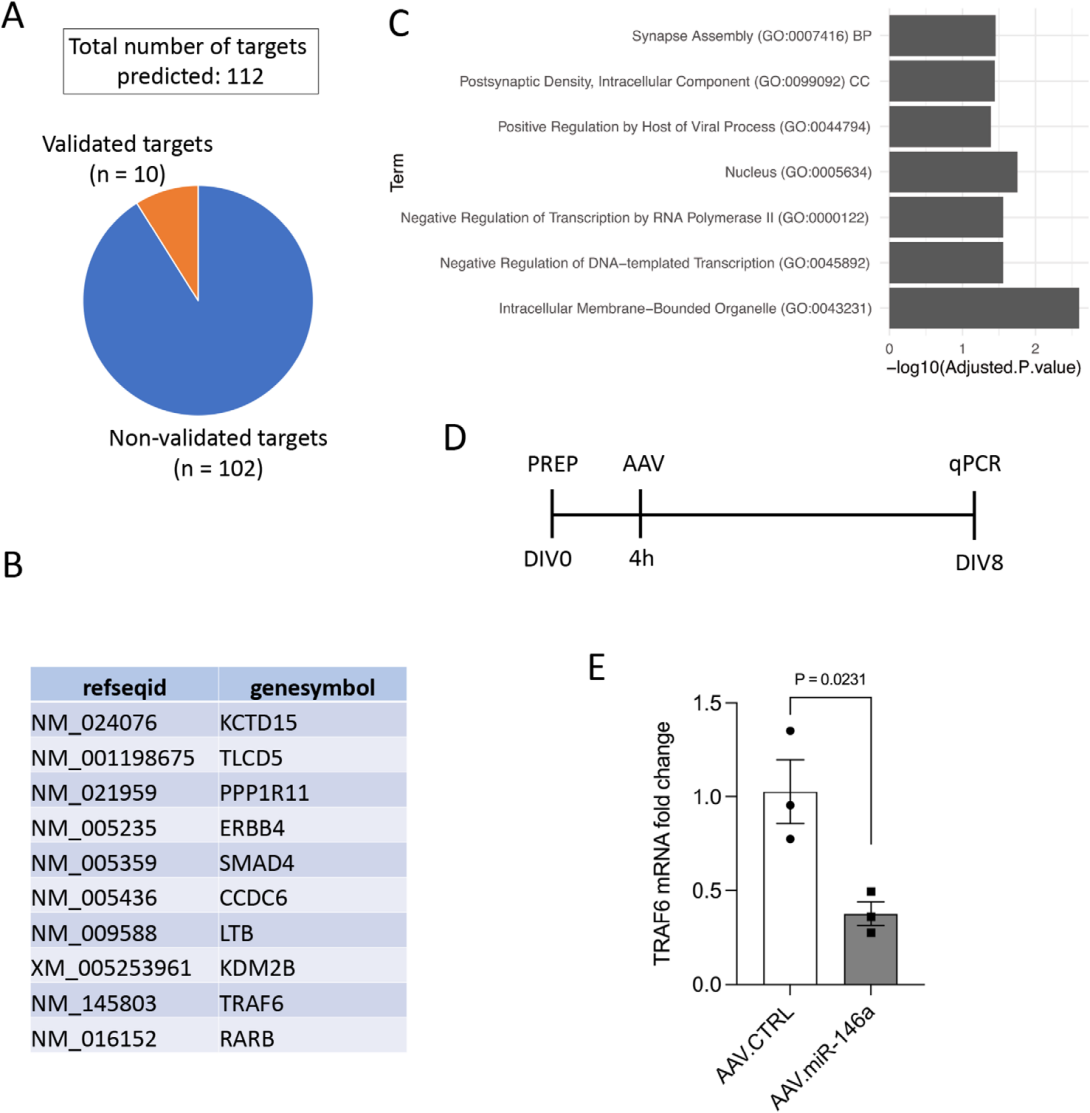
TRAF6 is a target of miR‐146a in cortical neurons. (A) Target prediction results, revealing a total of 112 predicted targets for hsa‐miR‐146a‐5p—of which 10 have been experimentally validated (B). (C) GO terms significantly (adjusted *p* value < 0.05) enriched for the 112 predicted targets of hsa‐miR‐146a‐5p. (D) Experimental design for the assessment of gene expression. PREP, preparation of E18 cortical neurons; AAV, viral transduction; DIV, days in vitro; qPCR, RNA extraction and qPCR analyses. (E) Expression of TRAF6 in primary cortical neurons after transduction with either AAV.CTRL or AAV.miR‐146a. Data represented as single data points and mean ± SEM (*n* = 3 independent cultures). Two‐tailed unpaired *t*‐test, *t* = 3.583, *p* = 0.0231.

MicroRNAs (miRNAs) are evolutionarily conserved, post‐transcriptional regulators of gene expression that play essential roles in diverse biological processes including cellular growth, proliferation, differentiation, metabolism, and organismal development (Ameres and Zamore 2013). To gain insight into the molecular mechanisms underlying the effects observed in this study, we performed target prediction analyses for the mature human strand hsa‐miR‐146a‐5p. A total of 112 putative targets were computationally predicted based on seed‐sequence complementarity and interaction probability (Sticht et al. 2018) (Figure 6A). Among these, 10 targets with prior experimental validation were extracted from the miRWalk database and included in the analysis (Figure 6A,B). Gene Ontology analysis of the predicted targets showed significant enrichment (adjusted *p* < 0.05) for terms primarily related to synaptic function and transcriptional regulation (Table S2). Specifically, top‐ranking categories included key terms like “Synapse Assembly,” “Postsynaptic Density, Intracellular Component,” “Nucleus,” “Negative Regulation of Transcription by RNA Polymerase II,” and “Negative Regulation of DNA‐templated Transcription” (Figure 6C). One of the most recurrent genes across these enriched categories was tumor necrosis factor receptor‐associated factor 6 (TRAF6), appearing in five of the seven significant terms. TRAF6 is a well‐established miR‐146a target (Taganov et al. 2006; Hou et al. 2009) and functions as a multifunctional signaling adaptor, playing key roles in inflammatory cascades (Dou et al. 2018), which is involved in many neurodegenerative diseases (Alemán‐Villa et al. 2025). To examine whether TRAF6 is regulated in our experimental paradigm, primary cortical neurons were transduced with either AAV.miR‐146a or AAV.CTRL, and TRAF6 mRNA levels were assessed by qPCR on DIV8 (Figure 6D). AAV.miR‐146a significantly reduced TRAF6 expression, producing a 62% decrease compared with AAV.CTRL (Figure 6E). Together, these results indicate that TRAF6 is a downstream target of miR‐146a in this experimental context, warranting further investigation into its potential role in the effects observed in this model.

## 4 Discussion

In this study, we explored whether AAV‐mediated overexpression of miR‐146a in primary cortical neurons could enhance neurite and axon regeneration. Our findings demonstrate that AAV.miR‐146a significantly promotes neurite outgrowth and arborization and, importantly, facilitates neurite and axonal regrowth in injury models. Target prediction and functional enrichment analyses further indicate that miR‐146a regulates gene networks associated with synaptic function and transcriptional control. Consistent with these predictions, we observed a marked reduction in TRAF6 expression following AAV.miR‐146a treatment.

Traumatic injuries to the CNS, such as spinal cord injury (SCI), often result in severe and irreversible deficits due to the limited regenerative capacity of mature CNS neurons. In contrast, neurons in the developing CNS and those of the adult PNS retain the ability to regenerate axons, largely because of a permissive transcriptional program, suggesting that molecules active in these contexts may serve as targets to enhance axon repair in the adult CNS. Among the regulators of gene expression, miRNAs have emerged as key modulators of CNS development, for instance, miR‐146a loss impairs the differentiation of radial glial cells, neurogenesis, and neurite extension (Fregeac et al. 2020). It is also enriched in dorsal root ganglion axons and promotes axonal growth in vitro (Jia et al. 2016). Conversely, reduced miR‐146a levels have been reported in the circulation of SCI patients (Paim et al. 2019) and in spinal cord tissue from rodent models of SCI (He et al. 2018; Tan et al. 2018). These observations led us to test whether AAV‐mediated overexpression of miR‐146a in CNS neurons in vitro could modulate neurite and axon growth and regeneration.

We first examined the effects of AAV.miR‐146a on neurite extension and branching and observed a significant increase in both the area of neurite immunostaining and the complexity of neurite arborization in transduced cortical neurons. Notably, similar findings have been reported in human neural stem cells, where miR‐146a overexpression drives neuronal differentiation and enhances neurite outgrowth and branching (Nguyen et al. 2018). These results are consistent with the idea that miR‐146a may activate intrinsic growth programs that support neurite extension. Such effects could reflect the regulation of transcriptional or signaling pathways that coordinate cytoskeletal dynamics, synaptic assembly, or other processes essential for neurite development and connectivity.

Although AAV.miR‐146a promoted neurite outgrowth, this effect does not necessarily imply enhanced regenerative capacity. To address this, we next assessed its impact in injury paradigms. In a mechanical scratch assay, AAV.miR‐146a transduction significantly increased neurite regeneration. Because this model does not distinguish axons from dendrites, we employed a microfluidic platform to selectively lesion axons and directly evaluate their regrowth. Consistently, AAV.miR‐146a increased the number of regenerating axons, reinforcing the idea that miR‐146a directly supports axonal repair. Although this analysis provides a robust endpoint assessment of axonal regeneration, it does not capture dynamic parameters of regrowth such as growth rate, directional persistence, or retraction frequency. Live‐cell imaging approaches would be required to determine whether miR‐146a modulates these aspects of intrinsic axonal growth behavior. Together, our results align with previous work showing that miR‐146a promotes axonal growth in peripheral neurons (Jia et al. 2016), broaden the understanding of miR‐146a as a regulator of neuronal repair and highlight this miRNA as a promising therapeutic target for CNS injuries such as SCI. Supporting this notion, exosomes enriched in miR‐146a derived from human umbilical cord mesenchymal stem cells reduce lesion size and improve motor function after SCI in rats (Lai et al. 2022), although whether these effects involve direct enhancement of motor axon regeneration remains unclear. Similarly, in peripheral neuropathy, administration of miR‐146a mimics restores sciatic nerve fiber morphology, suggesting additional neuroprotective roles (Liu et al. 2017).

To further understand the mechanisms behind the effects of AAV.miR‐146a, we performed bioinformatic and GO analyses to evaluate the genes regulated by miR‐146a. Axonal regeneration requires a massive regulation in gene expression (Ribas and Costa 2017), and we found, interestingly, that miR‐146a is predicted to regulate the expression of genes involved in transcription. Moreover, terms related to synaptic function were also significantly enriched. These findings suggest that miR‐146a may influence gene networks associated with transcriptional regulation and synaptic processes, both of which are relevant for neuronal plasticity and regeneration. However, the functional relevance of these predicted pathways in the context of axonal regeneration remains to be experimentally determined. Among the enriched categories, TRAF6, a well‐validated target of miR‐146a (Taganov et al. 2006), emerged as a frequent hit. Indeed, we found that TRAF6 is downregulated in cortical neuron culture after AAV.miR‐146a transduction. This observation supports the notion that TRAF6 is responsive to miR‐146a modulation in our experimental system.

TRAF6 is a key mediator of inflammatory signaling, acting downstream of both the TNF receptor and the interleukin‐1 receptor/toll‐like receptor (IL‐1R/TLR) superfamilies to regulate cell death, survival, and stress responses across multiple pathways (Kobayashi et al. 2004; Wu and Arron 2003). Injury to the CNS, such as SCI, elicits a strong inflammatory response that contributes to tissue damage and functional deficits (Chio et al. 2021). Because miR‐146a suppresses TRAF6 signaling and thereby attenuates inflammation (Taganov et al. 2006), it is conceivable that miR‐146a may influence inflammatory signaling pathways following CNS injury. Nevertheless, the extent to which TRAF6 modulation contributes to the regenerative effects observed here remains unresolved. More recently, TRAF6 has been implicated in axonal injury, where activation of neuronal p75^NTR recruits TRAF6 to engage the TAK1/JNK cascade, ultimately causing abnormal axonal morphology and impaired axonal transport (Liu et al. 2024). Because efficient axonal transport is critical for regeneration, it is plausible that miR‐146a promotes axonal regrowth by downregulating TRAF6, thereby preserving or enhancing transport. However, direct evidence linking miR‐146a–dependent TRAF6 regulation to axonal transport or regeneration is currently lacking, and future studies will be required to determine whether this pathway plays a functional role in mediating the regenerative effects of miR‐146a.

In conclusion, our findings identify miR‐146a as a potent enhancer of axonal regeneration in CNS neurons and provide initial mechanistic insights into its potential modes of action. Despite these promising findings, several limitations of this study should be acknowledged. First, all experiments were performed in primary cortical neuron cultures, which do not fully replicate the cellular diversity, extracellular environment, and complex circuitry of the injured CNS in vivo. Second, although AAV.miR‐146a enhanced neurite and axonal regrowth, we did not evaluate whether these structural changes translate into functional synaptic connectivity. Moreover, while TRAF6 emerged as a prominent downstream target, definitive validation of its involvement in axonal regeneration will require targeted loss‐ and gain‐of‐function studies. Finally, the safety of sustained AAV‐mediated delivery of miR‐146a requires careful assessment in future in vivo studies. Taken together, these results support miR‐146a as a promising modulator of intrinsic neuronal growth capacity, whereas underscoring the need for future in vivo and mechanistic studies to determine whether its potential effects on axonal transport, synaptic remodeling, and neuroinflammation translate into functional recovery.

## Author Contributions

**V. U. S. Matos:** writing – original draft, investigation, methodology, formal analysis, data curation, validation, visualization. **R. A. Almeida:** methodology, investigation, validation, visualization, formal analysis. **C. G. Ferreira:** methodology, investigation, validation, visualization, formal analysis. **M. T. R. Alves:** methodology, investigation, validation, visualization, formal analysis. **M. P. Braga:** methodology, investigation, validation, visualization, formal analysis. **M. C. Silva:** methodology, investigation, validation, visualization, formal analysis. **T. F. da Silva:** investigation, methodology, validation, visualization. **P. P. G. Guimarães:** methodology, investigation. **F. M. Soriani:** methodology, investigation. **P. Caramelli:** methodology, investigation, writing – review and editing. **M. R. Costa:** methodology, investigation, writing – review and editing, formal analysis. **U. Michel:** methodology, investigation, writing – review and editing. **V. T. Ribas:** conceptualization, investigation, funding acquisition, writing – review and editing, supervision, data curation, project administration, resources.

## Funding

This research was funded by Research Supporting Foundation of Minas Gerais State (FAPEMIG) grant numbers (APQ‐00218‐17, RED‐00187‐22, BPD‐00704‐22, APQ‐01857‐23, and APQ‐04985‐23), National Council for Scientific and Technological Development (CNPq) grant numbers (440498/2022‐8, 401811/2024‐7, and 406089/2024‐8). The Histochemical Society and Society for Neurochemistry (CDG). Paulo Caramelli receives research support by CNPq, Brazil.

## Conflicts of Interest

The authors declare no conflicts of interest.

## Supporting information


**Table S1:** Predicted targets for the mature form of human miR‐146a (hsa‐miR‐146a‐5p).

**Table S2:** Gene Ontology (GO) enrichment analysis for the 112 predicted miR‐146a targets.

## Data Availability

The data that supports the findings of this study are available in Supporting Information of this article.
